# Supplementary material for: Role of Chondroitin Sulfate Proteoglycan 5 in Steroid-Induced Cataract
Source: Cells. 2023 Jun 23;12(13):1705. doi: 10.3390/cells12131705 (PMC10340432; doi:10.3390/cells12131705)
Supplement: Supplementary file 1 [file cells-12-01705-s001.zip › cells-2439461-supplementary.pdf]

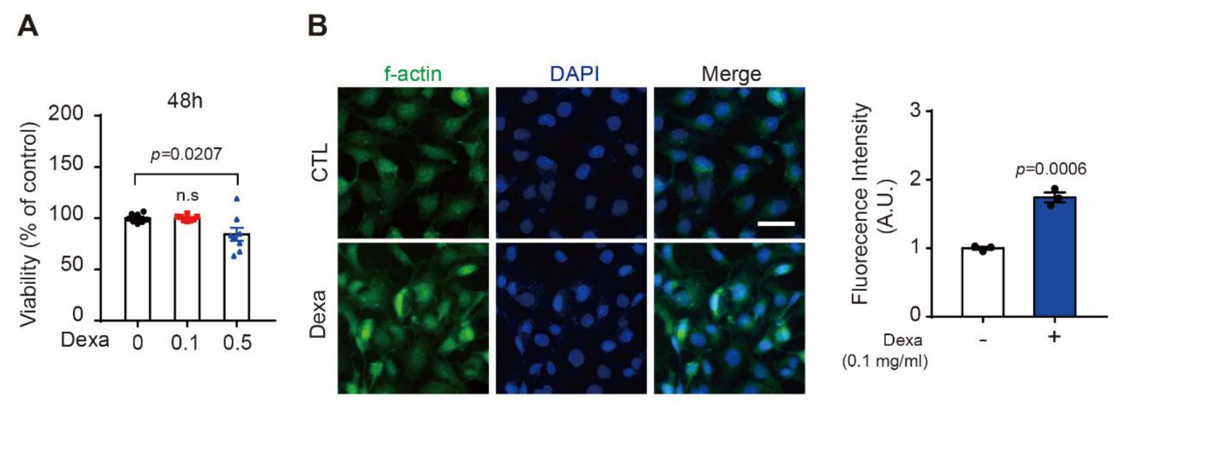

Figure S1. Effect of Dexa on viability of HLE-B3 cells. HLE-B3 cells were cultured in medium with various concentration of Dexa (0.1, 0.5 mg/ml) for 48 h. (A) The effect of Dexa on the activity of mitochondrial dehydrogenases in HLE-B3 cells was measured by the CCK-8 assay (n=8). The viability of control cells was set to 100%. Viability was represented as a percentage of control cells. $p < 0.05$ was the significance threshold. (B) The effect of Dexa on the cell morphology was detected by F-actin staining (Green). DAPI was used as nuclear marker (Blue). Scale bar, 25 μm. Ratio of protein expression determined using ImageJ software (n=3). Data are represented as means ± S.E.M

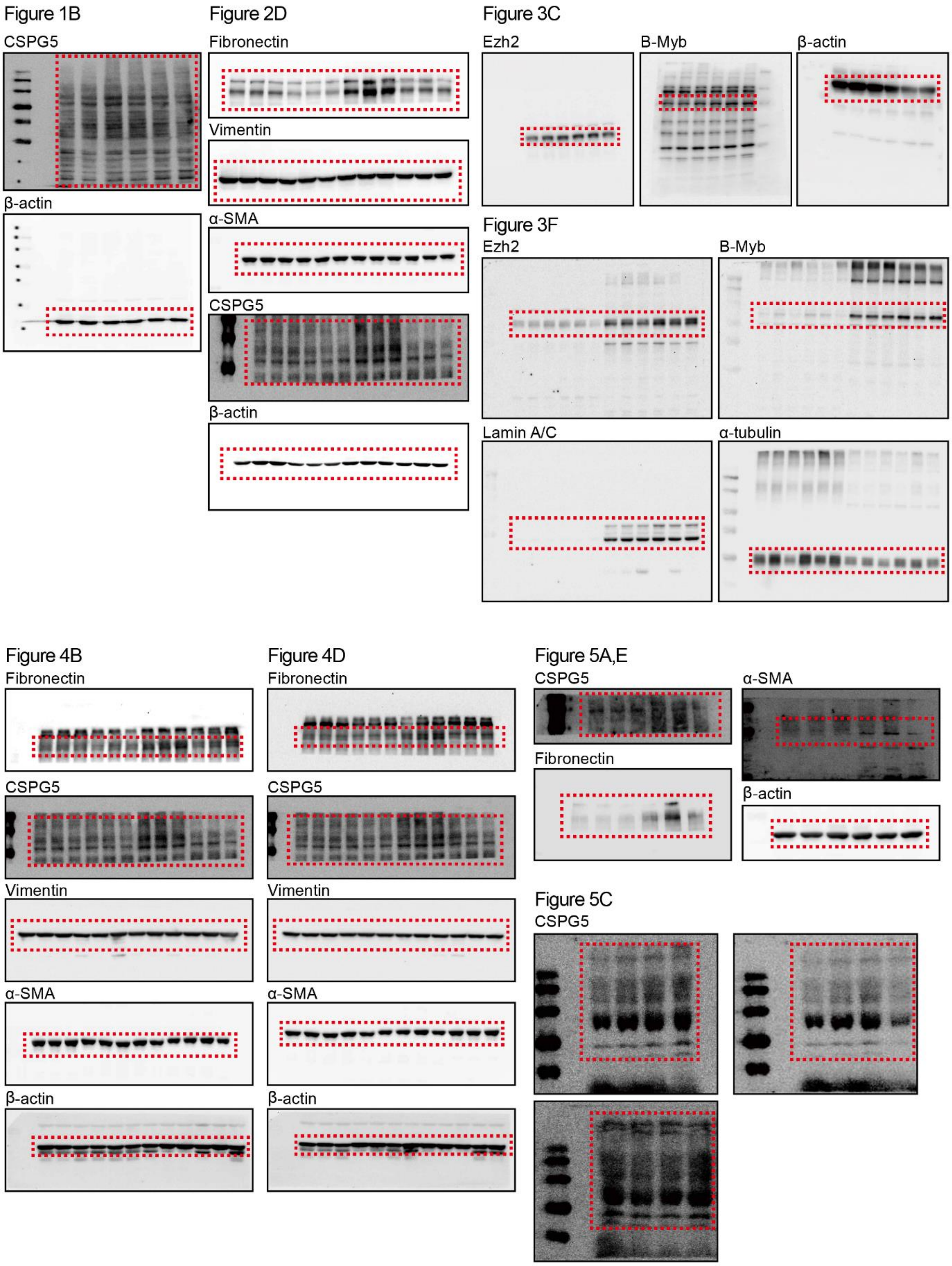

Figure S2. Original full western blot image of the manuscript

Table S1. Primary sequences used for mRNA expression analysis

	Gene	Primer	Sequences (5'-3')
1	*Cspg5*	Forward	AAG CTG CGT AGG ACC AAC AA
		Reverse	AGC ACT AGG ATC ATC ATT TGG GT
2	*Ezh2*	Forward	TGG TGC TGA AGC CTC AAT GT
		Reverse	GTA TCC ACA TCC TCA GCG GG
3	*Mybl2*	Forward	CAT GAG GAG AAC CGC ACT GA
		Reverse	TCC ACT GCT TTG TGC CAT ACT

Table S2. Clinical Characteristics of Patients with Steroid Induced Cataract

Patient No.	Sex	Age	BCVA (Log-MAR)	IOP (mmHg)	LOCS III Grade				Reason for Steroid Use
					NO	NC	C	P	
1	Male	59	1.3	15	2	2	1	2	VKH syn-drome
2	Female	82	1	15	4	4	3	2	Nodular scleritis
3	Male	45	1.3	19	3	3	1	2	Chronic uveitis
4R	Male	56	0.1	13	3	3	2	2	Kidney trans-planta-tion
4L	Male	56	0.2	16	3	3	1	2	Kidney trans-planta-tion
5	Male	70	0.8	10	4	4	1	2	Adrenal insuffi-ciency
6	Female	60	1.3	13	4	4	2	4	Eosino-philic granu-lomato-sis with polyan-gitis
7R	Male	60	0.2	11	3	3	1	3	Chronic derma-titis
7L	Male	60	0.3	12	3	3	1	2	Chronic derma-titis
8	Female	70	0.3	19	3	3	3	3	Chronic uveitis
9	Male	45	0.2	14	3	3	2	2	Kidney trans-planta-tion
10	Female	56	1.3	16	3	3	2	2	Chronic uveitis

BCVA=best corrected visual acuity; IOP=intraocular pressure; L=Left; R=Right

Table S3. Clinical Characteristics of patients with Age-Related Cataract

Patient No.	Sex	Age	BCVA (LogMAR)	IOP (mmHg)	LOCS III Grade			
					NO	NC	C	P
1	Female	71	0.5	12	3	3	4	0
2	Female	72	0.2	9	4	4	3	0
3R	Female	55	0.5	14	3	3	1	0
3L	Female	55	0.7	15	3	3	1	0
4	Male	65	0.4	8	4	4	2	1
5	Male	75	0.7	14	3	3	3	1
6R	Male	79	0.8	14	5	5	3	1
6L	Male	79	2	10	5	5	3	1
7R	Female	73	0.5	11	3	3	1	1
7L	Female	73	1	15	4	4	2	1
8	Female	56	0.7	20	4	4	2	0
9	Female	78	0.7	14	6	6	2	1
10	Female	73	0.5	16	3	3	3	1
11R	Female	73	0.7	18	3	3	3	0
11L	Female	73	0.7	16	3	3	3	0
12	Male	72	0.8	14	3	3	1	0
13R	Male	68	0.5	11	5	5	3	0
13L	Male	68	1.6	13	5	5	3	0
14	Male	74	0.5	14	4	4	3	1
15R	Male	54	0.7	14	4	4	4	1
15L	Male	54	0.7	14	4	4	4	0
16	Male	62	0.5	14	3	3	3	1
17	Female	65	2	17	4	4	1	1
18	Female	76	0.7	12	4	4	3	1
19	Female	70	0.3	13	3	3	2	0
20	Male	69	0.4	17	4	4	4	1
21R	Male	77	0.2	12	5	5	3	0
21L	Male	77	0.1	11	5	5	2	0
22	Male	39	1	16	3	3	3	0
23	Male	75	0.4	19	4	4	2	0
24	Female	64	0.2	13	3	3	5	0
25R	Male	60	0.7	11	3	3	1	0
25L	Male	60	0.7	12	3	3	1	0
26	Male	72	0.1	19	3	3	3	1
27	Male	74	0.8	16	4	4	1	0
28R	Male	68	0.5	12	4	4	2	0
28L	Male	68	0.7	14	4	4	2	1
29	Female	85	0.7	17	4	4	4	0
30	Female	76	0.7	18	4	4	2	1
31	Female	62	0.3	15	3	3	3	0

32R	Female	63	0.5	10	3	3	4	0
32L	Female	63	0.8	13	3	3	2	1
33	Female	69	2	11	4	4	5	1
34	Female	53	1.6	15	5	5	3	1
35	Female	55	0.1	12	3	3	4	1
36	Female	67	0.7	13	3	3	3	1
37	Male	65	0.7	18	4	3	1	0
38	Female	71	0.7	16	4	4	2	0
39R	Male	79	0.5	18	4	4	3	0
39L	Male	79	0.8	15	4	4	3	0

BCVA=best corrected visual acuity; IOP=intraocular pressure; L=Left; R=Right

Supplementary information

Figure S1. Effect of dexa on viability of HLE-B3 cells

Figure S2. Original full western blot images of the manuscript

Table S1. Primary sequences used for mRNA expression analysis

Table S2. Clinical characteristics of patients with steroid-induced cataract

Table S3. Clinical characteristics of patients with age-related cataract
